# A global database on land use and management change effects on soil KMnO4-oxidisable organic carbon (POXC)

**DOI:** 10.1038/s41597-025-05976-9

**Published:** 2025-10-27

**Authors:** Cécile Chéron-Bessou, Damien Beillouin, Alexis Thoumazeau, Lydie Chapuis-Lardy, Tiphaine Chevallier, Julien Demenois, Paul N. Nelson

**Affiliations:** 1 CIRAD, UMR ABSys, ELSA Group, F-34398 Montpellier, France; 2 ABSys, Univ Montpellier, CIRAD, Montpellier, France; 3 JCU, College of Science and Engineering, QLD-, 4878 Smithfield, Australia; 4 CIRAD, UPR HortSys, F-34398 Montpellier, France; 5 HortSys, Univ Montpellier, CIRAD, Montpellier, France; 6 IRD, UMR Eco&Sols, Montpellier, France; 7 CIRAD, UPR AIDA, F-34398 Montpellier, France; 8 AIDA, Univ Montpellier, CIRAD, Montpellier, France

**Keywords:** Environmental sciences, Plant sciences

## Abstract

Soil carbon transformation is vital for ecosystem functions like food production and climate regulation. While soil organic carbon is a key soil health indicator, its sensitivity to management changes is debated. Alternative indicators, such as permanganate-oxidisable carbon (POXC), are being explored. This database compiles 10,068 comparisons of soil POXC content from 284 peer-reviewed studies published up to 2023, covering 45 countries and 63 land use types, including arable land, grassland, agroforestry, and forests. Most studies focused on arable land (*n* = 7,809), examining input changes (*n* > 500) and tillage intensity (*n* > 200). The most studied land-use changes were grassland conversion to arable land (n = 324) and vice versa (n = 261). The dataset includes rich metadata on geographical context, soil types, key properties (pH, clay content), POXC protocols, and data quality scores. This resource supports scientific and policy discussions on POXC’s potential as a practical indicator for improving land use and soil health management.

## Background & Summary

Understanding and preserving soil health is critical to ensuring its continued ability to support life. Soils are at the core of most provisioning and regulating ecosystem services^1–3^. Despite its crucial role in both the ecosystem and the economy, soil degradation not only continues but is accelerating^4,5^. Human activities, such as deforestation, unsustainable agricultural practices, and urbanization, degrade soil health. Worldwide, at least 33% of croplands are moderately or highly degraded^6,7^. Land degradation affects 3.2 billion people, and results in an economic loss of approximately 10% of annual global gross product. These numbers stress that “an urgent step change in effort is needed to prevent irreversible land degradation and to accelerate the implementation of restoration measures”^8^. Both scientific research and policy discussions emphasise that achieving this goal requires standardised methodologies and regulations for monitoring, reporting and verifying (MRV) soil measurements that are sensitive to management practices^9–12^.

Soils are multifunctional, and it is impossible to maximize the provision of all ecosystem services simultaneously^13,14^. In agricultural soil management, trade-offs are unavoidable —whether between different services or across spatial and temporal scales— making their analysis, assessment and valuation challenging^13–15^. The provision of ecosystem services is driven, among other factors, by carbon transformation^16–18^. Soil carbon transformation is a continuous process driven by living organisms, influencing a wide range of ecosystem services, from food production to climate regulation. As a result, soil organic matter and embedded carbon have been central to many studies on soil health. In recent years, soil organic carbon (SOC) storage has also attracted increasing attention in relation to climate change mitigation strategies^19^. Various studies and databases on SOC have been developed^20–22^, and SOC has been used as one of the proxy indicators for soil health^23–25^ or to assess links between soil quality and agronomic outputs such as crop yields^26^. However, authors have also concluded that SOC may lack sufficient sensitivity to management practices to effectively guide soil health protection and land restoration strategies^27,28^. SOC comprises a diverse range of organic compounds with varying functions, mineral associations, and turnover rates, including highly stable compounds with long residence times. Therefore, SOC has limited utility for detecting rapid changes in soil quality or identifying functional shifts^29–31^, highlighting the need for more sensitive indicators.

Labile or active carbon pools represent a significant fraction of SOC, typically accounting for 10–20% of SOC, and play a key role in soil microbial activity and nutrient cycling. By definition, such pools have a rapid turnover, as they are more readily available to the soil food web as an energy source^32–34^. Changes in pool size may hence indicate variations in the activity of soil organisms driven by changes in soil management^30,35,36^. Permanganate oxidisable carbon (POXC) is one of the labile carbon pools quantified through oxidation by potassium permanganate (KMnO_4_). First measurements of POXC date back to 1987, but the most widely used protocols were established in 1995^37^, followed by further adjustments^38–41^, with the POXC acronym first introduced in 2003^42^. Several studies have shown that POXC is more sensitive to management changes than SOC, suggesting that POXC can be a valuable metric for quickly tracking management-induced changes in soil organic matter^33,43–47^. Moreover, POXC measurement relies on a cost-effective, straightforward protocol, which has contributed to its popularity as a soil health indicator^48,49^. Nevertheless, other studies have pointed to discrepancies among protocols^50–52^ and questioned the direct links between chemical oxidisability, biological availability, and their relationship to soil processes^49,53^. There is still a critical knowledge gap regarding the nature of the organic materials identified by the KMnO_4_ method, and how to interpret the sensitivity of POXC to changing management practices compared to other SOC parameters^38,40,54^. This database^55^ aims at providing comprehensive POXC datasets and metadata to facilitate further investigations on how POXC relates to soil management and health.

**Fig. 1:**
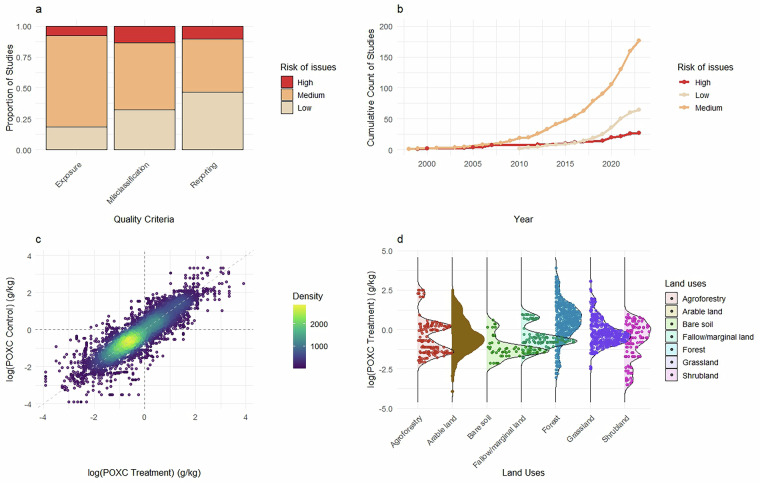
Overview of the POXC database V2 showing the study quality appraisal (**a**) and final score with tendencies along publication years (**b**), as well as a quantitative display of POXC measurements according to treatment and control (**c**), and over the various studied land uses (**d**).

This database^55^ provides a worldwide comprehensive and quantitative assessment of available datasets on the effects of major soil use and management interventions on POXC. It contains 10,068 quantified effect sizes, representing changes in POXC due to land use or land management changes, compared to a control. Where applicable, we considered both the original controls of the studies and/or compared parallel treatments against each other within the same studies as they also inform management changes. Data were published in 284 studies across 45 countries. We provide the locations of the primary studies (WGS84 datum), the type of interventions, applied protocols, and expert-cross-checked information on soil types, offering an opportunity to identify knowledge gaps. Through an iterative process, we defined archetypes for the various interventions in order to cover the main land uses, forestry managements and agricultural practices encountered across the studies. These archetypes were necessary to harmonise the assessment of land uses and management practices across the studies. In total, we covered 63 archetypes including 53 for agricultural management (embedding grassland and agroforestry), 4 for forest plantation management, 4 for natural vegetation stands, then fallow/marginal land and bare soil. Finally, to guide future work, we also identified transparency and quality issues based on a harmonised quality appraisal grid aggregating three criteria into a final quality score. This database is intended to advance the scientific and policy debate on the interest of using POXC as a tool to help and guide improved land use and management practices aimed at improving or maintaining soil health. In particular, it can support the refinement of characterisation factors within life cycle assessment as a component of soil health assessment. An overview of the database content is shown in Fig. 1.

## Methods

### Search process

The data collection followed Reporting Standards for Systematic Evidence Syntheses (ROSES) guidelines v3.2018^56^, and adhered to the gold standard of systematic review methods^57,58^. Our methodology is based on a repeatable paper selection procedure, provides a complete list of references, and ensures dataset availability.

**Fig. 2:**
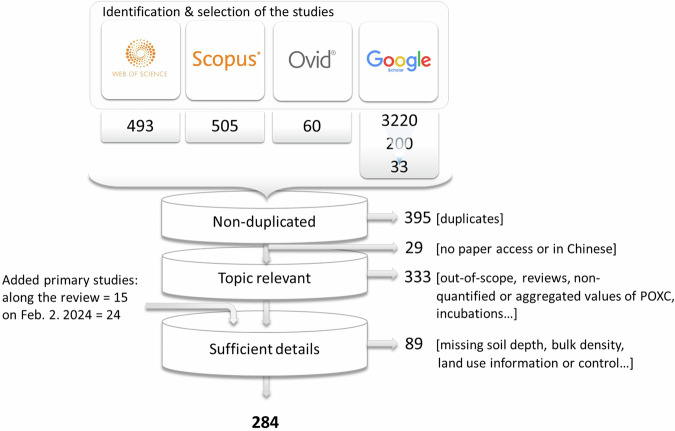
Identification and selection of the studies. For Google Scholar: 3,220 were the search total results; 200 were displayed on the first 10 pages that are the relevant ones; and 33 were the pre-selected studies remaining after rejection of the clearly out-of-scope search results. Added primary studies consisted in i) 15 primary studies with consistent POXC measurements identified through digging into meta-analysis and syntheses; and ii) 24 studies identified through the same literature search updated on February 2nd. 2024.

The literature search was initially conducted on February 03, 2023 and updated on February 02, 2024 (Fig. 2), and aimed to identify relevant peer-reviewed articles without language restriction. The following searching equation was used: “Soil AND carbon AND (permanganate OR POXC OR KMnO_4_)”, applied to the “topic words” (titles, abstracts and keywords). The search was carried out in 4 databases: Web of Science (WoS) core collection; Scopus; OVID; and Google Scholar. In Google Scholar, only the first 200 search results in English, ranked by relevance, were reviewed, as this engine is highly precise primarily within the top-ranked results^59^. In addition to the database searches, 15 primary studies or datasets identified in meta-analyses and systematic syntheses within the initial corpus were manually added during the review process.

No restriction was applied regarding the year of publication, except for Google Scholar, where the search was limited to studies published between 2003 and 2023. This constraint aimed at reducing the number of duplicates potentially added through Google Scholar, while still incorporating the most prolific publication period (Fig. 1a). All climatic zones and countries were considered. We prioritised sensitivity over specificity, meaning the search strategy aimed to collect the largest selection of potentially relevant studies at the cost of retrieving a larger number of non-relevant studies. This strategy increased the duration of the screening step and the number of final rejected studies. In total, 1,091 studies were identified, including 696 unique records, which were compiled as candidates for inclusion (Fig. 2).

### Article screening

All identified articles were screened based on their abstract and material and methods sections. When necessary, the full text was reviewed. The inclusion and exclusion criteria were defined using the PICOC framework: Population (i), Intervention (ii), Comparator (iii), Outcomes (iv), Context (v). For a study to be included, the following criteria had to be met:

i. **Effect on what?** The primary study examined the amount of *in situ* soil carbon that reacted with potassium permanganate (KMnO_4_) following a published protocol (protocols were recorded);
ii. **Effect of what?** The primary study analysed the effect of land use changes, encompassing shifts in land use type (natural or more or less exploited) or shifts in agricultural and forestry land management practices. Industrial or urban land uses were not included in the study. Studies using KMnO_4_ for bioremediation, rather than for assessing soil carbon stock related to a specific land use or management, were also excluded. Additionally, studies lacking any information on land use type or management were not considered;
iii. **Effect compared to what?** The change in land use or land management was compared to a control in a similar pedoclimatic context;
iv. **What is extracted?** The primary study provided the mean POXC concentration (in g.kg^−1^) over a given soil depth, along with a measure of dispersion (*e.g*., standard deviation (SD) or standard error of the mean (SE or SEM)), and the number of replicates for both the control and the treatments. Studies that expressed POXC in units other than g.kg^−^ were included only if sufficient information (*e.g*., bulk density) was available for conversion to g.kg^−1^.
v. **In which contexts is data extracted?** POXC measurements should be done in undisturbed soil to reflect actual *in situ* soil functioning. Therefore, lab incubations or measurements on chemically pre-treated soils were excluded.

The exclusion criteria were cross-checked by two authors on a subset of the recorded studies and refined where necessary. Each study was then screened by a single author, and discussions were held among co-authors to resolve any uncertainties regarding specific studies. Ultimately, the article authors were contacted directly via emails and Research Gate messages to shed some light on uncertainties or missing information, yielding a response rate of 40% (10 out of 25 inquiries). All final extractions were reviewed by the lead author (*see* Data validation).

Finally, POXC data were extracted from 284 studies and included in the database (Fig. 2). The rejected studies were compiled in an exclusion sheet, with reasons for their exclusion.

### Characterisation of the studies and extracted information

For each study, we documented author names, year of publication, title, journal name, abstract, keywords, DOI and URL when available. Additionally for each included study, we further extracted the following meta-data: KMnO_4_ protocol reference, location measurement name and/or GPS coordinates, mean annual precipitation and temperature (when reported), soil type and some initial soil variables such as SOC, pH, and clay content (if available), the type of field trial, number of replicates and number of POXC measurement dates.

Some studies presented results for different contexts, or different experimental set-up. All information was extracted as independent experiment, and resulted in multiple entries in the *C_RETAINED.STUDIES_POXC_DB.v1.1.csv* file. Similarly, some studies presented experimental data for different treatments against a control plot. When comparisons between these plots were conducted on the same soil type, consistent soil management histories, and identical protocols, all possible combinations of comparisons were included in the database, even if they were not explicitly presented in the original paper. This led to multiple entries in the *D_EFFECT.SIZES_POXC_DB.v1.1.csv* file.

**Fig. 3:**
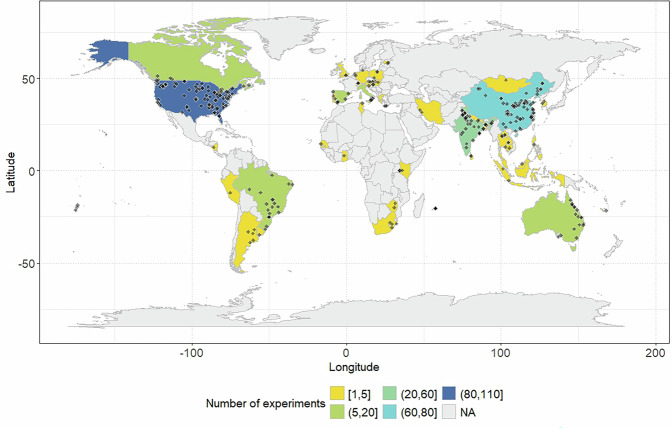
World map indicating the location of experiments where POXC data were recorded at country level.

**Fig. 4:**
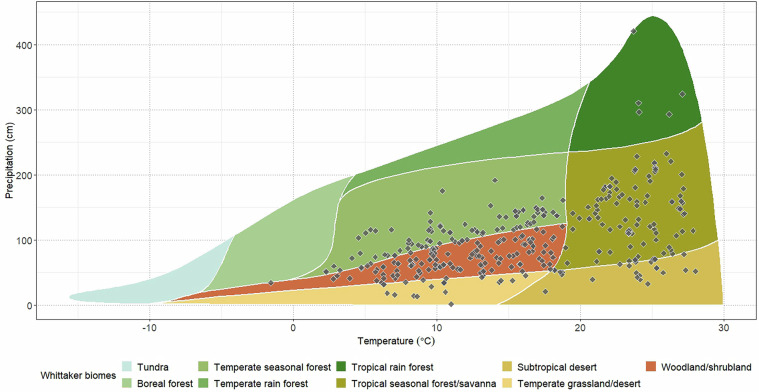
Whittaker plot showing the biomes represented by POXC recorded data points based on local mean annual precipitation and temperature over 1970–2020.

Coordinates were harmonised and, when necessary, converted to the WGS84 projection, using the free tool “Online converter to all coordinate systems” (https://coordinates-converter.com/en). Our database encompasses data from 45 countries, with highest numbers of experiments in the United States, followed by China, India, and Australia, Brazil and Canada. In contrast, Africa and Europe were the least investigated regions (Fig. 3). Based on the coordinates and information on historical mean annual precipitation and temperature from World Clim^60^ (https://www.worldclim.org/data/worldclim21.html), we plotted the recorded data points on a Whittaker biome plot. This analysis reveals that both tropical and temperate biomes (except for the seasonal forest) are well represented in the dataset, while boreal and tundra biomes are notably underrepresented (Fig. 4).

Mean POXC values per treatment and corresponding replicate numbers, at specified soil depth, were collected manually from tables or using the free online tool “Extracttable” (https://www.extracttable.com/), and from figures using another free online tool “WebPlot Digitizer software” (https://automeris.io/). Information was reported under various terms including POXC, permanganate oxidisable carbon (POC), labile carbon (LC), active carbon (AC), or reactive organic carbon (ROC), among others. For consistency, all these measurements are hereafter referred to as POXC.

We collected data on the precision of the POXC means, mainly using standard deviation (SD) or standard error of the mean (SEM). In cases where the nature of the dispersion value was unspecified, we assumed it to be SEM by default to avoid underestimating variability, while also keeping track of the added uncertainty. In a few cases, we also estimated SD from 95% confidence intervals^61^ (*n* = 12 studies). In other cases where dispersion information was not provided but results of statistical tests were available (*e.g.*, letters indicating significance from Tukey’s test or Least Significant Difference values), we used the free EX-TRACT tool_1.2 to estimate pooled standard deviations^62^ (*n = *75 studies) (Supplementary Table 1). For the estimated SD obtained from this tool, we recorded the maximum pooled SD value to avoid overconfidence in the results. Furthermore, in cases of uneven replicate numbers among treatments and controls, assumptions were made to maximize pooled SD and mitigate error underestimation. This global strategy may help balance bias and account for underrepresented studies that show no effect^63^. Eventually, for the last studies lacking any dispersion information, we calculated the median coefficients of variation within subsets of studies with similar soil types, soil depths and land uses using only data where the original SD was available. We then applied these median coefficients of variation to the means of treatments to compute missing SD values.

We developed a comprehensive land use classification designed to analyse effects in ways that facilitate practical application. Land uses and management practices can be described in varying ways according to the studies, which may hamper any cross-cutting analysis. To address this issue, we classified land uses and land management practices into classes and subclasses. Building on the IPCC^20,64^ classification, we first established higher-order classes: cropland, forest, grassland, wetland, settlement and other land. Settlement and other land categories were excluded as they fell outside the study’s scope, while shrubland, fallow/marginal land and bare soil were added. Wetland was not treated as a separate class to avoid overlapping with soil type classification. To further refine our classification, cropland was subdivided into annual crops, perennial crops and agroforestry systems. By default, “arable land” refers exclusively to annual crops, while specific terms such as “arable land, perennial” and “agroforestry” are used for the latter categories.

Within each land use class, we then defined sub-classes to capture the diversity of managements practices. The sub-classes were iteratively refined during the review process in order to cover all the case studies, while maintaining sufficient number of occurrences within each sub-class for meaningful analysis. Arable land classes were not further disaggregated by crop type, due to the large variety of crops. The only exception was rice, which was covered in many studies and required specific practices related to irrigation mode. Forest types and forest plantation managements were also further differentiated. In total, we defined and applied 63 combinations of land use classes and sub-classes, also called archetypes (Table 1).

**Table 1:** Archetypes detailed by high-order land use classes and management sub-classes.

High-order land use classes	Management types	Management sub-classes	Archetypes numbers
arable land arable land, perennial crop agroforestry forest forest, plantation grassland shrubland	Not specified or not relevant for the land use comparison	unspecified	7
bare soil	None	None	1
fallow/marginal land	None	None	1
agroforestry	Input management	▪ no input ▪ low inputs ▪ medium inputs ▪ high inputs	4
agroforestry	Intercropping (spatial)	▪ with legumes ▪ without legumes	2
arable land	Soil physical disturbance	▪ no tillage ▪ intermediate intensity tillage ▪ high intensity tillage	3
arable land	Water management Specific to rice	▪ Irrigated ▪ non-irrigated ▪ flooded ▪ non/alternatively flooded	4
arable land	Rotations (temporal)	▪ with legumes/cover crop ▪ without legumes/cover crop	2
arable land	Intercropping (spatial)	▪ intercropping ▪ no intercropping	2
arable land	Integrated management systems	▪ conventional ▪ organic ▪ integrated/agroecological	3
arable land arable land, perennial crop grassland	Input management	▪ no input ▪ low mineral inputs ▪ medium mineral inputs ▪ high mineral inputs ▪ low organic inputs ▪ medium organic inputs ▪ high organic inputs ▪ mineral and organic inputs	24
forest	Natural stand Semi-natural stand	▪ primary undisturbed ▪ secondary or regrowth	2
forest, plantation	Intensity of intervention given production objectives	▪ low productivity ▪ medium productivity ▪ high productivity	3
grassland	Intercropping (spatial)	▪ intercropping ▪ with legumes ▪ without legumes	3
grassland	Grazing Intensity	▪ grazed ▪ ungrazed	2
Total number of archetypes			63

Archetypes were defined for each treatment and each control based on the primary management factor investigated. The data dictionary provides all the details about these archetypes. In some cases, a single treatment (or control) could fit into multiple archetypes due to the combination of different management practices. For these cases, other investigated factors were recorded as bias risks. In some cases, one treatment needed to be duplicated and attributed different archetypes in order to treat management factors separately. In such cases, records kept track of duplicates and ensured consistent land use or land management comparisons.

**Fig. 5:**
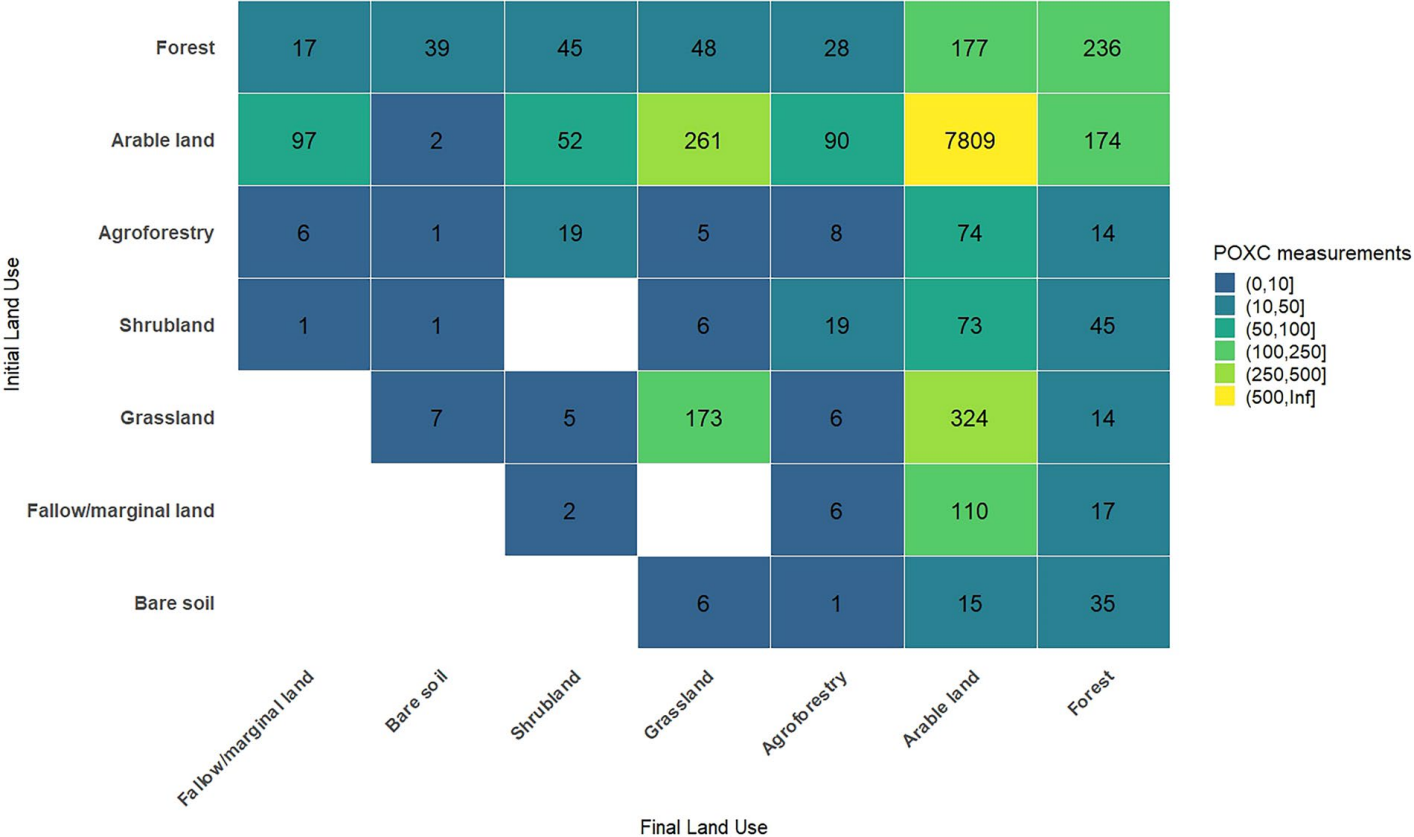
Matrix of covered land use and land use changes in the POXC database V2.

**Fig. 6:**
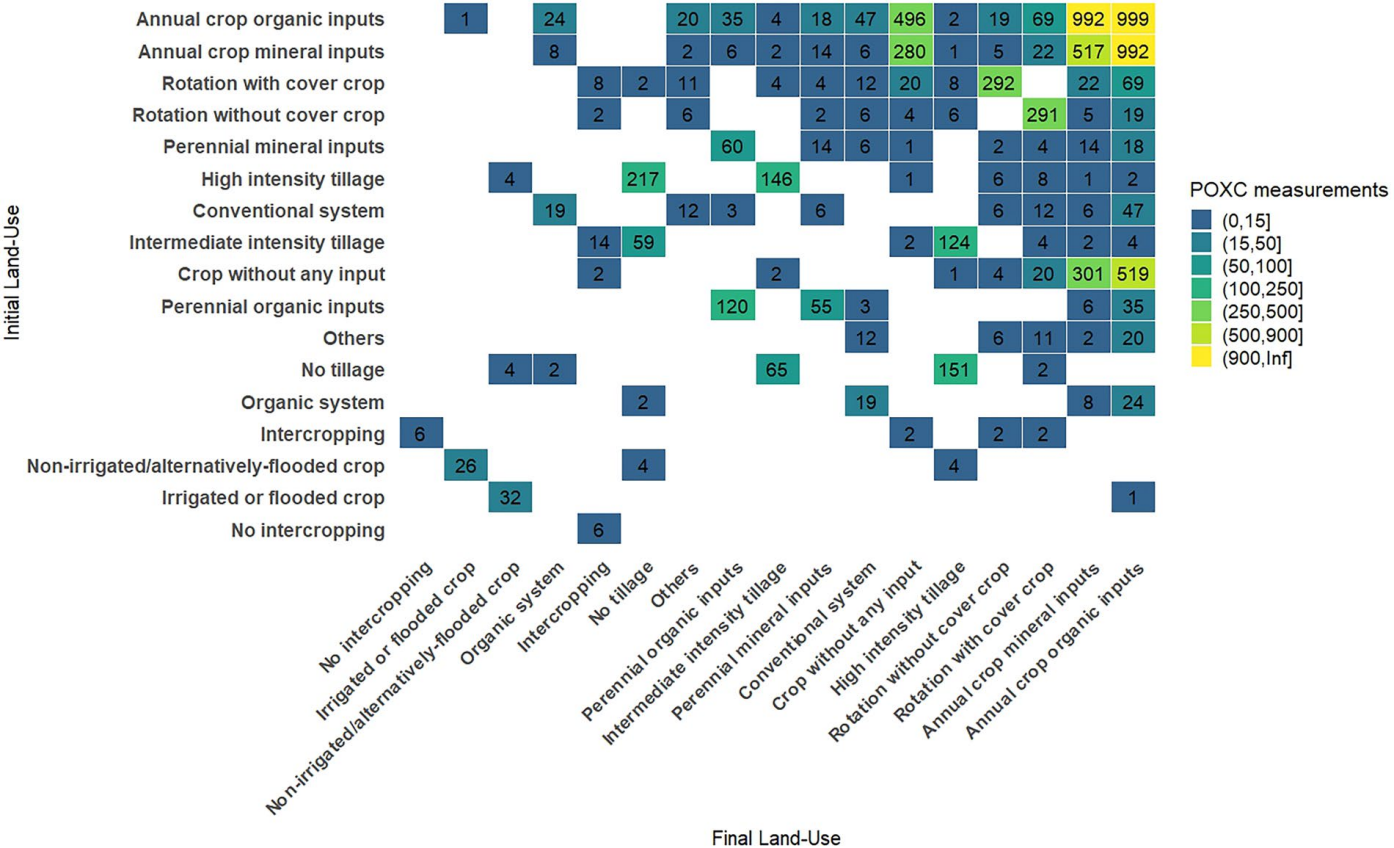
Matrix of covered land management and management changes for arable land in the POXC database V2.

Overall, this database contains 10,068 comparisons of soil POXC content across various land uses and managements practices. Most studies focused on management changes within arable land (*n* = 7,809) (Fig. 5). Among these studies, change in input types and application rates were the most frequently studied practices (*n* > 500), followed by tillage intensity and cover crop (both *n* > 200) (Fig. 6). The most frequently studied land-use changes were from grassland to arable land (*n* = 324) and vice-versa (*n* = 261) (Fig. 5). There was a similar number of studies on deforestation to arable land (*n* = 177) and afforestation from arable land to forest (*n* = 174) (Fig. 5).

Finally, we paid a meticulous attention to soil types and properties, which are, intrinsically, closely related to soil health and soil carbon pools. We compiled all soil information provided by the authors and, based on the recorded coordinates, completed it with information from the Harmonized World Soils Database version 2.0^65^ (HWSD2), World Soil Information referred to as ISRIC maps (soilgrids), and expert knowledge. ISRIC maps were downloaded from the webpage (https://files.isric.org/soilgrids/latest/data/). We focused on maps of clay content and pH (H_2_O) at 0-5 cm and 5-15 cm soil depths to check and, where necessary, complete missing author-reported data. For each site, we then compiled the best estimates for the reference soil group (RSG) following the harmonised World Reference Base^66^, the clay content (%) and the pH (depending on the method used by the authors, *see* Supplementary Table 2). We also proposed a grouping of these three variables based on their suitability or main constraints for biological activities to help with the data treatment (Supplementary Table 2). Despite all our efforts, there remain some uncertainties related to clay content and pH, which one should bear in mind when analysing these co-variables together with POXC measurements.

## Data Records

This database^55^ adheres to the FAIR principles, ensuring that all information is accessible, robust and transparent for reuse and integration into future analytical frameworks (Supplementary Table 3).

The files are grouped into two categories: i) The database file starting with the number 1, is the one file to be directly used to work with the data, as it contains all the useful variables for any analysis; ii) Files starting with a capital letter are the ones providing complementary information to delve into the individual studies and the database establishment.

All files are provided in .csv format except for the E_QUALITY_APPRAISAL_TOOL_POXC_DB.v1.0 Excel spreadsheet due to formatted decision trees as well as the supplementary material provided as a pdf. The “README” file outlines the study authors, context and lists all associated files as follows:

- 1_EFFECT.SIZES_POXC_DB.v1.1.csv: This file contains all quantitative comparisons between the treatments and the controls. Each row corresponds to a POXC measurement from a specific pedo-climatic location and soil depth; one article may yield multiple rows if it includes measurements from several locations or soil depths;
- A_STUDIES_POXC_DB.v1.1.csv: This file provides the complete list of the 699 studies identified in the February 2023 search, excluding duplicates (except for 3 discovered later), plus 24 additional studies identified in February 2024. The metadata include details for each study;
- B_REJECTED.STUDIES_POXC_DB.v1.1.csv: This file lists the 454 studies discarded due to one or more exclusion criteria. If a study ‘inclusion was uncertain, it was initially retained and further assessed;
- C_RETAINED.STUDIES_POXC_DB.v1.1.csv: This file lists all the retained experimental sites and provides detailed metadata on the POXC protocol, geographical coordinates, soil type, field trial characteristics, and the number of POXC measurements. From the 284 studies, a total of 372 experimental sites were identified, as some studies included multiple locations;
- D_QUALITY.APPRAISAL.TOOL_POXC_DB.v1.0.xlsx: This file details the study quality appraisal approach developed for the database;
- E_QUALITY.SCORES_POXC_DB.v1.0.csv: This file lists the detailed quality scores by study, based on meticulous quality review process described in the “Technical validation” section;
- F_ADDED.STUDIES_POXC_DB.v1.0.csv: This file includes all studies extracted from secondary sources (*e.g*., some initial references were reviews listing other studies). In total, 53 studies were identified: 17 were already present in the list from the original search, 5 were inaccessible, and 15 were retained after applying the selection criteria. DOIs or URLs are provided where available;
- G_DATA.DICTIONARY_POXC_DB.v1.1.csv: This file defines all terms and archetypes used in the database.
- H_SUPPLEMENTARY.MAT_POXC_DB.v1.1.pdf: This file contains three tables with supplementary information to the datapaper on (1) the estimation of dispersion based on the free EX-TRACT tool_1.2^62^_,_ (2) the grouping of some soil variables, and (3) the applied FAIR principles.

## Technical Validation

The data extraction process was carefully reviewed to minimise errors and ensure the highest possible level of accuracy. Data quality assurance and control involved three complementary steps: 1) using plots to visually identify potential errors in both in POXC measurements and geographical coordinates; 2) reviewing extracted data from the retained studies to verify consistency through iterative discussions between the lead author and each co-author; and 3) conducting an overall data quality screening using a robust critical appraisal framework.

For the first step, following the initial data extraction, we used RStudio (2024.04.2) with R-4.3.0 to carefully inspect for extreme values, potential unit discrepancies and other inconsistencies. We also plotted the geographical coordinates to identify and correct potential errors.

For the second step, the authors discussed a total of 198 potential issues (*e.g*., uncertainties regarding POXC measurements or replicates, or ambiguities in selecting the archetypes). These detailed discussions led to the exclusion of 20 studies —representing 522 POXC measurements — that had been initially retained. These discarded studies are included in the total number of rejections provided in Fig. 2. In a few cases (*n* = 11 studies), not all POXC measurements (*e.g*., treatments or soil depths) were extracted, primarily for three reasons: i) some data could not be extracted from figures due to insufficient resolution or details; ii) some data were not relevant to land use or management analysis; or iii) some data were redundant with already extracted data.

Finally, for the third step, we implemented a robust critical appraisal strategy to identify potential sources of bias and enhance the reliability of the dataset. Following the Collaboration for Environmental Evidence (CEE) approach but adjusted to agronomy, we defined three key bias risk criteria, each supported by a decision tree. To ensure objectivity in the critical appraisal process, the lead author conducted a consistent review of all retained studies and collaborated with the primary reviewers whenever ambiguities arose. The key bias risk criteria were as following:

i. **Misclassification risks:** These risks refer to cases where land use classification was ambiguous—due to unclear treatments or land use definitions, land uses not matching the defined archetypes, imprecise control definitions, or potential confounding effects;
ii. **Length/exposure risks:** These risks address situations where the experimental duration was unclear, where the control might not have matched the age of the treatments plot, or where exposure was likely insufficient to ensure the origin of observed effects;
iii. **Outcome reporting risks:** These risks focus on uncertainty regarding POXC unit, soil depth, or data extraction from figures; or cases where dispersion metrics were not provided and had to to be estimated.

Final quality scores were categorised as “Low”, “Medium”, and “High” risk of issues. The overall score was determined based on the most frequent risk level (*i.e*., at least two out of three criteria). When each level occurred once, the score was set to “Medium risk of issues”. The only exception was for cases where treatments and controls occurred on different soil types, indicating a significant risk based on expert knowledge. In these cases (*n* = 5 studies), the scores were set to “High risk of issues”. Both initial and final reviewers’ comments were recorded to document the main concerns and justify the decision. These quality scores are intended to help to weight studies in meta-analyses, and to adjust the mean overall calculated effect for varying degree of bias.

## Usage Notes

To our knowledge, this is the most comprehensive database^55^ of soil POXC measurements related to changes in land use and land management practices so far. It is designed as a valuable resource for both scientists and decision-makers to analyse the effectiveness and uncertainty associated with various land use strategies and land management practices aiming at preserving or improving soil health, with a particular focus on the soil carbon transformation function. In addition to POXC data, the database includes a wide range of complementary variables, such as pH, clay content, and crop yields where available, allowing for deeper analysis of POXC sensitivity as a soil health indicator under varying conditions.

Crucially, this database is designed to be dynamic and can be easily updated with new data as the number of studies on this topic continues to grow. We encourage researchers and practitioners to contribute relevant studies or data not yet included. Submissions can be sent directly to the corresponding author to support the ongoing expansion and utility of this resource.

## Supplementary information

Supplementary material to the datapaper

## Data Availability

All data files are available in the CIRAD repository Version 2, and can be accessed at: 10.18167/DVN1/78A2II.

## Code availability

R code was used to:

• Extract HWSD2 data by coordinates, compile and check soil types and initial properties.

• Figures and maps

• Data initial check

The code to upload data and draw maps and figures is made available on GitHub: https://github.com/bessou-web/POXC_Database
