# Supplementary material for: A global database on land use and management change effects on soil KMnO4-oxidisable organic carbon (POXC)
Source: Sci Data. 2025 Oct 27;12:1697. doi: 10.1038/s41597-025-05976-9 (PMC12559398; doi:10.1038/s41597-025-05976-9)
Supplement: Supplementary file 1 — Supplementary material to the datapaper [file 41597_2025_5976_MOESM1_ESM.pdf]

A global database on land use and management change effects on soil KMnO4-oxidisable organic carbon (POXC)

Cécile Chéron-Bessou[1,2,3*], Damien Beillouin[4,5], Alexis Thoumazeau[1,2], Lydie Chapuis-Lardy[8], Tiphaine Chevallier[8], Julien Demenois[6,7], Paul N. Nelson[3]

Affiliations

1. CIRAD, UMR ABSys, ELSA Group, F-34398 Montpellier, France
2. ABSys, Univ Montpellier, CIRAD, Montpellier, France
3. JCU, College of Science and Engineering, QLD-4878 Smithfield, Australia
4. CIRAD, UPR HortSys, F-34398 Montpellier, France
5. HortSys, Univ Montpellier, CIRAD, Montpellier, France
6. CIRAD, UPR AIDA, F-34398 Montpellier, France
7. AIDA, Univ Montpellier, CIRAD, Montpellier, France
8. IRD, UMR Eco&Sols, Montpellier, France

corresponding author(s): Cécile Chéron-Bessou (cecile.bessou@cirad.fr)

Supplementary material to the datapaper

Content: 3 tables

Supplementary material Table 1: Standard deviations estimated based on published statisitical information and the EX-TRACT tool by Acutis, M. et al. EX-TRACT: An excel tool for the estimation of standard deviations from published articles. Environ. Model. Softw. 147, 105236 (2022)

Studies ID	***Set***	***SDValue***	***SDMin***	***SDMax***	***Method***	***n***	***DFE***	***Notes***
38_Benbi_et_2015b	1		0,231809	0,338155	LETTER - Tukey	22	105	a, ab, bc, c, ab
106_Culman_et_2010	0-10cm	0,107895	0,10594	0,110	P(F)	5	4	
106_Culman_et_2010	10-20cm	0,07075	0,069057	0,072	P(F)	5	4	
106_Culman_et_2010	20-40cm	0,019331	0,01864	0,020	P(F)	5	4	
106_Culman_et_2010	40-60cm	0,020853	0,020721	0,021	P(F)	5	4	
176PRT_Galvao_et_2005	1		0,207585	infinite	LETTER - Tukey	133	264	All means are not significantly different so SDMAX is set to infinite
173_Frene_et_al_2020	1		0,006745	0,029986	LETTER - LSD	6	15	
173_Frene_et_al_2020	2		0,007476	infinite	LETTER - LSD	6	15	All means are not significantly different so SDMAX is set to infinite
173_Frene_et_al_2020	3		0,030473	infinite	LETTER - LSD	6	15	All means are not significantly different so SDMAX is set to infinite
173_Frene_et_al_2020	4		0,010076	infinite	LETTER - LSD	6	15	All means are not significantly different so SDMAX is set to infinite
119_Datta_et_al_2010	1	0,085655	0,0828	0,08851	LSD	3	14	
121_de Figueiredo_et_2018	1		0,081733	0,127022	LETTER - Tukey	3	6	
123_de Moraes Sa_et_2014	1		0	0,140147	LETTER - LSD	3	6	All means are significantly different so SDmin is set to 0
123_de Moraes Sa_et_2014	3		0	0,150158	LETTER - LSD	3	6	All means are significantly different so SDmin is set to 0
123_de Moraes Sa_et_2014	4		0	0,155163	LETTER - LSD	3	6	All means are significantly different so SDmin is set to 0
123_de Moraes Sa_et_2014	5		0,220232	0,260274	LETTER - LSD	3	6	
127_Decker_et_2022	WREC 0-5cm		0,034314	0,041365	LETTER - Tukey	4	21	
127_Decker_et_2022	TVREC 0-5cm		0,039485	0,045596	LETTER - Tukey	4	21	
127_Decker_et_2022	TVREC 10-15cm		0,039485	0,046066	LETTER - Tukey	4	21	

Studies ID	***Set***	***SDValue***	***SDMin***	***SDMax***	***Method***	***n***	***DFE***	***Notes***
141_Didawat_et_2023	1		25,19462	26,94425	LETTER - Tukey	3	12	
141_Didawat_et_2023	2		12,94724	20,99552	LETTER - Tukey	3	12	
142_Diederich_et_2019	1		0,076638	0,083092	LETTER - LSD	4	15	
142_Diederich_et_2019	15-30		0,05889	0,095192	LETTER - LSD	4	15	
142_Diederich_et_2019	15-30		0,077445	0,086319	LETTER - LSD	4	15	
142_Diederich_et_2019	0-15		0,067764	0,104873	LETTER - LSD	4	15	
142_Diederich_et_2019	0-15		0,078251	0,078251	LETTER - LSD	4	15	
142_Diederich_et_2019	15-30		0,066151	0,089545	LETTER - LSD	4	15	
142_Diederich_et_2019	15-30		0,05889	infinite	LETTER - LSD	4	15	All means are not significantly different so SDMAX is set to infinite
142_Diederich_et_2019	0-15		0,074218	infinite	LETTER - LSD	4	15	All means are not significantly different so SDMAX is set to infinite
142_Diederich_et_2019	0-15		0,078251	infinite	LETTER - LSD	4	15	All means are not significantly different so SDMAX is set to infinite
142_Diederich_et_2019	15-30		0,065344	infinite	LETTER - LSD	4	15	All means are not significantly different so SDMAX is set to infinite
148_Dulazi_et_2022	0-15cm		0,148411	0,159405	LETTER - LSD	3	10	
15_Armstrong_et_1999	1	0,076954	0,074206	0,079702	LSD	3	10	
19_Auler_et_2020	0-10cm		0,449355	0,551964	LETTER - Tukey	3	6	
19_Auler_et_2020	10-20cm		0,176912	0,201679	LETTER - Tukey	3	6	
206_Gruver_2015	0-7.5cm		0,014456	0,031733	LETTER - Tukey	3	10	
206_Gruver_2015	0-7.5cm bis		0	0,171018	LETTER - Tukey	4	6	All means are significantly different so SDmin is set to 0
206_Gruver_2015	7.5-15cm		0,012446	0,094959	LETTER - Tukey	4	6	
223_Hendricks_et_2022	0-10cm		0,020755	0,063056	LETTER - Tukey	3	4	

Studies ID	*Set*	*SDValue*	*SDMin*	*SDMax*	*Method*	*n*	*DFE*	*Notes*
223_Hendricks_et_2022	20-30cm		0,003161	0,01213	LETTER - Tukey	3	4	
223_Hendricks_et_2022	30-40cm		0,001959	0,021545	LETTER - Tukey	3	4	
246_Jagadamma_et_2019	0-15cm		0,032267	infinite	LETTER - Tukey	4	6	All means are not significantly different so SDMAX is set to infinite
246_Jagadamma_et_2019	0-2.5cm		0,013829	0,165025	LETTER - Tukey	4	6	
246_Jagadamma_et_2019	2.5-5cm		0,065457	0,071449	LETTER - Tukey	4	6	
246_Jagadamma_et_2019	5-7.5cm		0,031346	infinite	LETTER - Tukey	4	6	All means are not significantly different so SDMAX is set to infinite
246_Jagadamma_et_2019	7.5-10cm		0,034572	infinite	LETTER - Tukey	4	6	All means are not significantly different so SDMAX is set to infinite
246_Jagadamma_et_2019	10-15cm		0,024431	infinite	LETTER - Tukey	4	6	All means are not significantly different so SDMAX is set to infinite
246_Jagadamma_et_2019	15-22.5cm		0,028119	infinite	LETTER - Tukey	4	6	All means are not significantly different so SDMAX is set to infinite
246_Jagadamma_et_2019	22.5-30cm		0,005071	infinite	LETTER - Tukey	4	6	All means are not significantly different so SDMAX is set to infinite
246_Jagadamma_et_2019	0-15cm		0,002666	infinite	LETTER - Tukey	4	3	All means are not significantly different so SDMAX is set to infinite
246_Jagadamma_et_2019	0-2.5cm		0	0,073767	LETTER - Tukey	4	3	All means are significantly different so SDmin is set to 0
246_Jagadamma_et_2019	2.5-5cm		0	0,024885	LETTER - Tukey	4	3	All means are significantly different so SDmin is set to 0
246_Jagadamma_et_2019	5-7.5cm		0	0,036883	LETTER - Tukey	4	3	All means are significantly different so SDmin is set to 0
246_Jagadamma_et_2019	7.5-10cm		0,029329	infinite	LETTER - Tukey	4	3	All means are not significantly different so SDMAX is set to infinite
246_Jagadamma_et_2019	10-15cm		0,008443	infinite	LETTER - Tukey	4	3	All means are not significantly different so SDMAX is set to infinite
246_Jagadamma_et_2019	15-22.5cm		0,024441	infinite	LETTER - Tukey	4	3	All means are not significantly different so SDMAX is set to infinite
246_Jagadamma_et_2019	22.5-30cm		0,019553	infinite	LETTER - Tukey	4	3	All means are not significantly different so SDMAX is set to infinite
246_Jagadamma_et_2019	Milan expe_0-15		0,044426	infinite	LETTER - Tukey	3	10	All means are not significantly different so SDMAX is set to infinite
246_Jagadamma_et_2019	Milan expe_0-2,5		0,037374	0,043368	LETTER - Tukey	3	10	

Studies ID	Set	SDValue	SDMin	SDMax	Method	n	DFE	Notes
246_Jagadamma_et_2019	Milan expe_2,5-5		0,021155	0,027854	LETTER - Tukey	3	10	
246_Jagadamma_et_2019	Milan expe_5-7,5		0,011635	0,021508	LETTER - Tukey	3	10	
246_Jagadamma_et_2019	Milan expe_7,5-10		0,020097	infinite	LETTER - Tukey	3	10	All means are not significantly different so SDMAX is set to infinite
246_Jagadamma_et_2019	Milan expe_10-15		0,033848	infinite	LETTER - Tukey	3	10	All means are not significantly different so SDMAX is set to infinite
246_Jagadamma_et_2019	Milan expe_15-22,5		0,029406	infinite	LETTER - Tukey	3	10	All means are not significantly different so SDMAX is set to infinite
246_Jagadamma_et_2019	Milan expe_22,5-30		0,024046	infinite	LETTER - Tukey	3	10	All means are not significantly different so SDMAX is set to infinite
246_Jagadamma_et_2019	Milan expe_30-45		0,025986	infinite	LETTER - Tukey	3	10	All means are not significantly different so SDMAX is set to infinite
246_Jagadamma_et_2019	Milan expe_45-60		0,01294	infinite	LETTER - Tukey	3	10	All means are not significantly different so SDMAX is set to infinite
249_Jensen_et_2019	LA		0	0,065457	LETTER - Tukey	4	6	All means are significantly different so SDmin is set to 0
256_Johnson_et_al_2021	TVREC: 0-5cm		0,072859	infinite	LETTER - Tukey	4	21	All means are not significantly different so SDMAX is set to infinite
256_Johnson_et_al_2021	TVREC: 5-10cm		0,034784	infinite	LETTER - Tukey	4	21	All means are not significantly different so SDMAX is set to infinite
256_Johnson_et_al_2021	TVREC: 10-15cm		0,068628	0,069098	LETTER - Tukey	4	21	
262_Kalisz_2012	Germany-dose	0,675292	0,675291	0,675292	P(F)	5	8	F-value=0.548
262_Kalisz_2012	Poland-dose	0,439211	0,439209	0,439213	P(F)	5	8	F-value=12.724 p(F)
262_Kalisz_2012	Estonia-dose	0,43606	0,436059	0,43606	P(F)	5	8	F-value=0.385
104_Crowell_et_2022	0-30cm		0,020451	infinite	LETTER - Tukey	3	6	All means are not significantly different so SDMAX is set to infinite
123_de Moraes Sa_et_2014	5-10cm.2002		0,125132	infinite	LETTER - LSD	3	6	All means are not significantly different so SDMAX is set to infinite
123_de Moraes Sa_et_2014	10-20cm.2002		0,090095	infinite	LETTER - LSD	3	6	All means are not significantly different so SDMAX is set to infinite
123_de Moraes Sa_et_2014	10-20cm.2005		0,280295	infinite	LETTER - LSD	3	6	All means are not significantly different so SDMAX is set to infinite
123_de Moraes Sa_et_2014	20-40cm.2005		0,160169	infinite	LETTER - LSD	3	6	All means are not significantly different so SDMAX is set to infinite

Studies ID	***Set***	***SDValue***	***SDMin***	***SDMax***	***Method***	***n***	***DFE***	***Notes***
123_de Moraes Sa_et_2014	20-40cm.2002		0,0951	infinite	LETTER - LSD	3	6	All means are not significantly different so SDMAX is set to infinite
127_Decker_et_2022	5-10cm TVREC		0,022093	infinite	LETTER - Tukey	4	21	All means are not significantly different so SDMAX is set to infinite
127_Decker_et_2022	5-10cm WREC		0,018332	infinite	LETTER - Tukey	4	21	All means are not significantly different so SDMAX is set to infinite
127_Decker_et_2022	10-15cm WREC		0,021623	infinite	LETTER - Tukey	4	21	All means are not significantly different so SDMAX is set to infinite
127_Decker_et_2022	15-30cm WREC		0,025853	infinite	LETTER - Tukey	4	21	All means are not significantly different so SDMAX is set to infinite
142_Diederich_et_2019	0-15cm Aug		0,07446	infinite	LETTER - LSD	4	15	All means are not significantly different so SDMAX is set to infinite
142_Diederich_et_2019	0-15cm Sept.		0,077929	infinite	LETTER - LSD	4	15	All means are not significantly different so SDMAX is set to infinite
142_Diederich_et_2019	15-30cm Sept.		0,065505	infinite	LETTER - LSD	4	15	All means are not significantly different so SDMAX is set to infinite
142_Diederich_et_2019	15-30cm Aug.		0,058971	infinite	LETTER - LSD	4	15	All means are not significantly different so SDMAX is set to infinite
148_Dulazi_et_2022	0-5cm		0,467221	infinite	LETTER - LSD	3	10	All means are not significantly different so SDMAX is set to infinite
148_Dulazi_et_2022	5-10cm		0,357287	infinite	LETTER - LSD	3	10	All means are not significantly different so SDMAX is set to infinite
148_Dulazi_et_2022	10-15cm		0,148411	infinite	LETTER - LSD	3	10	All means are not significantly different so SDMAX is set to infinite
138_Dhaliwal_et_al_2022	0-5cm		0,036259	0,046262	LETTER - LSD	4	9	
173_Frene_et_al_2020	0-15cm		0,007476	infinite	LETTER - LSD	6	15	All means are not significantly different so SDMAX is set to infinite
173_Frene_et_al_2020	15-30cm 2018		0,030473	infinite	LETTER - LSD	6	15	All means are not significantly different so SDMAX is set to infinite
173_Frene_et_al_2020	15-30cm 2019		0,010076	infinite	LETTER - LSD	6	15	All means are not significantly different so SDMAX is set to infinite
223_Hendricks_et_2022	10-20cm		0,015669	infinite	LETTER - Tukey	3	4	All means are not significantly different so SDMAX is set to infinite
223_Hendricks_et_2022	40-60cm		0,023092	infinite	LETTER - Tukey	3	4	All means are not significantly different so SDMAX is set to infinite
223_Hendricks_et_2022	60-80cm		0,035634	infinite	LETTER - Tukey	3	4	All means are not significantly different so SDMAX is set to infinite
223_Hendricks_et_2022	80-100cm		0,024294	infinite	LETTER - Tukey	3	4	All means are not significantly different so SDMAX is set to infinite

Studies ID	*Set*	*SDValue*	*SDMin*	*SDMax*	*Method*	*n*	*DFE*	*Notes*
273_Kinoshita_et_al_2017	0-6cm		0,018575	0,066598	LETTER - Tukey	4	9	control SOD not included in the test, SD from the other treatments taken
273_Kinoshita_et_al_2017	6-18cm		0,030354	0,049382	LETTER - Tukey	4	9	control SOD not included in the test, SD from the other treatments taken
273_Kinoshita_et_al_2017	18-30cm		0,035791	0,057084	LETTER - Tukey	4	9	control SOD not included in the test, SD from the other treatments taken
271_Kim_et_2022	0-20cm		0,024643	0,031932	LETTER - Tukey	3	14	
279_Kumar_et_2023	0-15cm		0,031562	0,038477	LETTER - Duncan	3	10	
279_Kumar_et_2023	15-30cm		0,045765	infinite	LETTER - Duncan	3	10	All means are not significantly different so SDMAX is set to infinite
279_Kumar_et_2023	30-60cm		0,008245	0,010259	LETTER - Duncan	3	10	
308_Li_et_2019	0-10cm		0,047078	0,059918	LETTER - Tukey	4	18	
308_Li_et_2019	10-20cm		0,064197	0,085597	LETTER - Tukey	4	18	
331_Lucas_Weil_2012	Beltsville		0	0,035447	LETTER - Tukey	4	3	All means are significantly different so SDmin is set to 0
331_Lucas_Weil_2012	Holtwood		0	0,049635	LETTER - Tukey	4	3	All means are significantly different so SDmin is set to 0
331_Lucas_Weil_2012	Keedsville		0	0,079271	LETTER - Tukey	4	3	All means are significantly different so SDmin is set to 0
331_Lucas_Weil_2012	Marlboro		0	0,078544	LETTER - Tukey	4	3	All means are significantly different so SDmin is set to 0
333_Lucas_et_2022	KSU 2016		0,00597	0,008798	LETTER - LSD	2	3	
333_Lucas_et_2022	KSU 2017		0,009427	0,01194	LETTER - LSD	2	3	
36_Benbi_et_2017	7.5-15cm	0,048862	0,048597	0,049128	LSD	3	8	CRD assumed
36_Benbi_et_2017	0-7.5cm	0,066389	0,066123	0,066654	LSD	3	8	CRD assumed
37_Benbi_et_2015a	0-7.5cm		0	0,053007	LETTER - Tukey	4	9	All means are significantly different so SDmin is set to 0
37_Benbi_et_2015a	7.5-15cm		0,040774	0,083361	LETTER - Tukey	4	9	Organic amendment
37_Benbi_et_2015a	7.5-15cmMIN		0,04757	infinite	LETTER - Tukey	4	9	All means are not significantly different so SDMAX is set to infinite

Studies ID	*Set*	*SDValue*	*SDMin*	*SDMax*	*Method*	*n*	*DFE*	*Notes*
37_Benbi_et_2015a	0-7.5cmMIN		0,017669	infinite	LETTER - Tukey	4	9	All means are not significantly different so SDMAX is set to infinite
374_Mazur_2015	Lessive_FI=33	0,041517	0,037365	0,045668	LSD	6	20	
374_Mazur_2015	Lessive_FII=167	0,02491	0,020758	0,029062	LSD	6	20	
374_Mazur_2015	Lessive_FIII=333	0,033213	0,029062	0,037365	LSD	6	20	
374_Mazur_2015	Brown_FI=33	0,02491	0,020758	0,029062	LSD	6	20	
374_Mazur_2015	Brown_FII=167	0,008303	0,004152	0,012455	LSD	6	20	
374_Mazur_2015	Brown_FIII=333	0,02491	0,020758	0,029062	LSD	6	20	
390_Mirsky_et_2008	Corn		0	0,018439	LETTER - Tukey	16	6	All means are significantly different so SDmin is set to 0
390_Mirsky_et_2008	Corn-soybean		0,003688	0,029963	LETTER - Tukey	16	6	
390_Mirsky_et_2008	Corn-alfalfa		0,015673	0,017517	LETTER - Tukey	16	6	
390_Mirsky_et_2008	Corn-oat-wheat-clover		0,011985	infinite	LETTER - Tukey	16	6	All means are not significantly different so SDMAX is set to infinite
409_Nayak_et_2009	C0		0,032398	0,135964	LETTER - Duncan	3	8	
409_Nayak_et_2009	C1		0,072231	0,096677	LETTER - Duncan	3	8	
412_Neogi_et_2014	rice PI		0	0,005921	LETTER - Duncan	3	2	All means are significantly different so SDmin is set to 0
412_Neogi_et_2014	rice T		0	0,008112	LETTER - Duncan	3	2	All means are significantly different so SDmin is set to 0
412_Neogi_et_2014	rice GF		0	0,00316	LETTER - Duncan	3	2	All means are significantly different so SDmin is set to 0
412_Neogi_et_2014	rice M		0	0,003957	LETTER - Duncan	3	2	All means are significantly different so SDmin is set to 0
412_Neogi_et_2014	maize M		0	0,011357	LETTER - Duncan	3	2	All means are significantly different so SDmin is set to 0
412_Neogi_et_2014	maize LI		0	0,023142	LETTER - Duncan	3	2	All means are significantly different so SDmin is set to 0
412_Neogi_et_2014	maize SK		0	0,01688	LETTER - Duncan	3	2	All means are significantly different so SDmin is set to 0

Studies ID	*Set*	*SDValue*	*SDMin*	*SDMax*	*Method*	*n*	*DFE*	*Notes*
412_Neogi_et_2014	maize CB		0	0,008995	LETTER - Duncan	3	2	All means are significantly different so SDmin is set to 0
412_Neogi_et_2014	cowpea LIA		0	0,015058	LETTER - Duncan	3	2	All means are significantly different so SDmin is set to 0
412_Neogi_et_2014	cowpea FL		0	0,010987	LETTER - Duncan	3	2	All means are significantly different so SDmin is set to 0
412_Neogi_et_2014	cowpea PF		0	0,005835	LETTER - Duncan	3	2	All means are significantly different so SDmin is set to 0
412_Neogi_et_2014	cowpea M		0	0,007372	LETTER - Duncan	3	2	All means are significantly different so SDmin is set to 0
42_Bhattacharyya_et_2012a	1		0	0,01902	LETTER - Duncan	3	6	All means are significantly different so SDmin is set to 0
444_Pearsons_2023	FT		0,002766	0,025814	LETTER - Tukey	4	6	
256_Johnson_et_al_2021	TVREC: 0-5cm		0,020239	infinite	LETTER - Tukey	4	21	All means are not significantly different so SDMAX is set to infinite
256_Johnson_et_al_2021	TVREC: 5-10cm		0,020661	infinite	LETTER - Tukey	4	21	All means are not significantly different so SDMAX is set to infinite
256_Johnson_et_al_2021	TVREC: 10-15cm		0,032045	infinite	LETTER - Tukey	4	21	All means are not significantly different so SDMAX is set to infinite
256_Johnson_et_al_2021	TVREC: 15-30cm		0,031624	infinite	LETTER - Tukey	4	21	All means are not significantly different so SDMAX is set to infinite
451_Peters_et_2020	1		0,034806	0,056225	LETTER - Tukey	3	8	
459_Prasad_et_2016	Main factor: tillage		0,044112	0,097046	LETTER - LSD	12	4	
459_Prasad_et_2016	Sub factor: ferti		0,050485	0,191845	LETTER - LSD	9	18	
46_Blair_et_1998	0-1cm		0	2,426159	LETTER - Duncan	3	4	All means are significantly different so SDmin is set to 0
46_Blair_et_1998	1-25cm		0,044112	infinite	LETTER - Duncan	3	4	All means are not significantly different so SDMAX is set to infinite
46_Ayr_Blair_et_1998	0-1cm		0	0,685662	LETTER - Duncan	5	8	All means are significantly different so SDmin is set to 0
46_Ayr_Blair_et_1998	1-10cm		0,205699	infinite	LETTER - Duncan	5	8	All means are not significantly different so SDMAX is set to infinite
46_Ayr_Blair_et_1998	10-25cm		0	infinite	LETTER - Duncan	5	8	All means are not significantly different so SDMAX is set to infinite
46b_Ball-Coelho_et_1993	t0: 0-7,5cm		0	0,11028	LETTER - Duncan	3	4	All means are significantly different so SDmin is set to 0

Studies ID	*Set*	*SDValue*	*SDMin*	*SDMax*	*Method*	*n*	*DFE*	*Notes*
46b_Ball-Coelho_et_1993	t1: 0-7,5cm		0,030878	infinite	LETTER - Duncan	3	4	All means are not significantly different so SDMAX is set to infinite
46b_Ball-Coelho_et_1993	t2: 0-7,5cm		0	0,101458	LETTER - Duncan	3	4	All means are significantly different so SDmin is set to 0
630_Whitbread_et_2000	crop vs fallow		0,040042	0,280295	LETTER - Duncan	3	6	
630_Whitbread_et_2000	stubble removal		0	0,122459	LETTER - Duncan	8	4	All means are significantly different so SDmin is set to 0
47c_Konboon_et_1999	litter input		0,025647	0,08583	LETTER - Duncan	3	10	
485_Romaniw_2015	0-5cm		0,285829	0,577155	LETTER - LSD	3	10	
485_Romaniw_2015	5-10cm		0,142915	0,192385	LETTER - LSD	3	10	
485_Romaniw_2015	10-20cm		0,219869	0,3353	LETTER - LSD	3	10	
24_Badagliacca_et_2020	0-5cm		0,111848	0,137361	LETTER - Duncan	30	174	n=30 pseudo-replicates, in fact 6 plots per land use and 5 samples per plot
24_Badagliacca_et_2020	5-30cm		0,035243	0,039246	LETTER - Duncan	30	174	n=30 pseudo-replicates, in fact 6 plots per land use and 5 samples per plot =. Issue with the letters at this depth, modified for more "b"
24_Badagliacca_et_2020	Moccasin		0,025757	0,040448	LETTER - LSD	4	9	
24_Badagliacca_et_2020	Bozeman		0,013177	0,026182	LETTER - LSD	4	6	
487_Rudrappa_et_al_2006	Fife		0	0,04047	LETTER - LSD	8	14	CRD chosen instead of the actual field-scale strip trial not available
487_Rudrappa_et_al_2006	0-15cm		0	0,064155	LETTER - Duncan	4	18	All means are significantly different so SDmin is set to 0
487_Rudrappa_et_al_2006	15-30cm		0,033657	0,032077	LETTER - Duncan	4	18	1,21 bc => 1,21 b
487_Rudrappa_et_al_2006	30-45cm		0,006108	0,012461	LETTER - Duncan	4	18	
489_Sainju_et_2021	Crops		0,054389	0,082521	LETTER - LSD	4	9	
490_Sainju_et_2021	crops			0,016017	LETTER - LSD	3	6	
490_Sainju_et_2021	N		0,001708	infinite	LETTER - LSD	3	2	

Studies ID	***Set***	***SDValue***	***SDMin***	***SDMax***	***Method***	***n***	***DFE***	***Notes***
497_Sepahvand_Feizian_2016	0-15cm		0,100038	0,152439	LETTER - Tukey	4	12	0,65c=>0,65b if not error
504_Sharma_et_2020	0-7,5cm		0,060775	0,062207	LETTER - Duncan	4	45	0,46abc=>ab not homogeneous among 3 occurrrences
504_Sharma_et_2020	7,5-15cm		0,044275	0,046734	LETTER - Duncan	4	45	0,39:cdef harmonised
508_Sheng_et_2015	0-20cm		0,43821	infinite	LETTER - Tukey	3	6	All means are not significantly different so SDMAX is set to infinite
508_Sheng_et_2015	20-40cm		0,118142	infinite	LETTER - Tukey	3	6	All means are not significantly different so SDMAX is set to infinite
508_Sheng_et_2015	40-60cm		0,113789	infinite	LETTER - Tukey	3	6	All means are not significantly different so SDMAX is set to infinite
508_Sheng_et_2015	60-80cm		0,114603	infinite	LETTER - Tukey	3	6	All means are not significantly different so SDMAX is set to infinite
508_Sheng_et_2015	80-100cm		0,225102	infinite	LETTER - Tukey	3	6	All means are not significantly different so SDMAX is set to infinite
513_Shrestha_et_2006	0-15cm_TEMP1998	0,495521	0,493019	0,498024	LSD	3	6	
513_Shrestha_et_2006	0-15cm_TEMP1999	0,230242	0,22774	0,232745	LSD	3	6	
513_Shrestha_et_2006	0-15cm_TEMP2000	0,760801	0,758298	0,763303	LSD	3	6	
513_Shrestha_et_2006	0-15cm_TROP1998	0,230242	0,22774	0,232745	LSD	3	6	
513_Shrestha_et_2006	0-15cm_TROP1999	0,240253	0,23775	0,242755	LSD	3	6	
513_Shrestha_et_2006	0-15cm_TROP2000	0,410432	0,407929	0,412935	LSD	3	6	
513_Shrestha_et_2006	15-30cm_TROP1998	0,155163	0,152661	0,157666	LSD	3	6	
513_Shrestha_et_2006	15-30cm_TROP1999	0,160169	0,157666	0,162671	LSD	3	6	
513_Shrestha_et_2006	30-60cm_TEMP1999	0,120126	0,117624	0,122629	LSD	3	6	
513_Shrestha_et_2006	30-60cm_TEMP2000	0,130137	0,127634	0,13264	LSD	3	6	further depths/crops NS
519_Singh_Benbi_2018b	0-7.5cm		0,019065	0,034086	LETTER - Duncan	3	16	
519_Singh_Benbi_2018b	7.5-15cm		0,023687	0,046797	LETTER - Duncan	3	16	404ab=>b; 363b=>c; 590bc=>d; 779d=>e

Studies ID	*Set*	*SDValue*	*SDMin*	*SDMax*	*Method*	*n*	*DFE*	*Notes*
55b_Messmer_et_2010	CH2		0,040774	0,054366	LETTER - Tukey	4	9	
55c	NOT USED				P(F)	4	3	CI95 used instead
566_Thorburn_et_2012	Abergowrie 0-2cm		0,395225	0,598823	STAR	4	6	
566_Thorburn_et_2012	Abergowrie 2-5cm		0,14522	0,220029	STAR	4	6	
566_Thorburn_et_2012	Abergowrie 5-10cm		0,068013	0,10305	STAR	4	6	
566_Thorburn_et_2012	Abergowrie 10-25cm		0,068013	0,10305	STAR	4	6	
566_Thorburn_et_2012	Ayr 0-2cm		0,645268	0,977675	STAR	4	6	
566_Thorburn_et_2012	Ayr 2-5cm		0,685702	1,038938	STAR	4	6	
566_Thorburn_et_2012	Ayr 5-10cm		0,413611	0,62668	STAR	4	6	
566_Thorburn_et_2012	Mackay 0-2cm		0,461407	0,699099	STAR	4	6	
566_Thorburn_et_2012	Woodford 0-2cm		0,505541	0,765968	STAR	4	6	other depths NS no way to calculate SD
576_Tirol-Padre_Ladha_2004	1997 0-20cm		0	0,250065	LETTER - Duncan	4	9	Replicates = 4 (control was 3) => larger SD taken
576_Tirol-Padre_Ladha_2004	1999 0-20cm		0,187548	0,750194	LETTER - Duncan	4	9	Replicates = 4 (control was 3) => larger SD taken
598_Verma_et_2010	SW	0,159889	0,157034	0,162745	LSD	3	14	CRBD
598_Verma_et_2010	MW	0,178031	0,174734	0,181327	LSD	4	14	split plot
598_Verma_et_2010	RW	0,257155	0,253858	0,260452	LSD	4	14	split plot
587_Umrit_et_2014	Alma		0,144273	infinite	LETTER - Tukey	3	6	All means are not significantly different so SDMAX is set to infinite
587_Umrit_et_2014	Beauchamp		0	0,957577	LETTER - Tukey	3	6	All means are significantly different so SDmin is set to 0
587_Umrit_et_2014	Medine		0,073131	0,373688	LETTER - Tukey	3	8	
587_Umrit_et_2014	Richeterre		0,264335	0,330161	LETTER - Tukey	3	8	

Studies ID	*Set*	*SDValue*	*SDMin*	*SDMax*	*Method*	*n*	*DFE*	*Notes*
587_Umrit_et_2014	Savannah		1,034504	1,178977	LETTER - Tukey	3	6	
600_Vieira_et_2007	Crops x 5		0,026556	0,228378	LETTER - Duncan	6	8	No replicate for control = letter chosen because same value as another crop
600_Vieira_et_2007	N ferti x 2		0	0,203379	LETTER - Duncan	15	10	All means are significantly different so SDmin is set to 0
607_Wang_et_2014	ferti		0,15031	0,22687	LETTER - LSD	3	12	
628_Wendling_et_2008	LU 10-20cm		0,078309	0,078309	LETTER - Tukey	4	21	1,32 ab=>a; 1,14bc=>b
628_Wendling_et_2008	LU 0-10cm		0,139215	0,156617	LETTER - Tukey	4	21	1,6bc=>b; 1,46bcd=>bc. 1,14dc=>cde
117_Das_et_2017	TREATMENT	0,022841	0,019986	0,025697	LSD	3	14	
86c_Lou_et_2011b	LU 0-20cm		0	0,071457	LETTER - LSD	3	10	All means are significantly different so SDmin is set to 0
77_Chan_et_2001	TREATMENT	0,175045	0,171919	0,178171	LSD	4	9	
75_Chakraborty_et_2019	Tillage				LSD	6	2	NOT USED, average calculated across treatments
698_Zou_et_2018	2011: 0-10cm		0,03949	0,041499	LETTER - Tukey	3	10	
698_Zou_et_2018	2012: 0-10cm		0,021367	infinite	LETTER - Tukey	3	10	All means are not significantly different so SDMAX is set to infinite
698_Zou_et_2018	2013: 0-10cm		0,037797	0,053135	LETTER - Tukey	3	10	
698_Zou_et_2018	2013: 10-20cm		0,026303	infinite	LETTER - Tukey	3	10	All means are not significantly different so SDMAX is set to infinite
698_Zou_et_2018	2012: 10-20cm		0,020344	infinite	LETTER - Tukey	3	10	All means are not significantly different so SDMAX is set to infinite
698_Zou_et_2018	2011: 10-20cm		0,03138	infinite	LETTER - Tukey	3	10	All means are not significantly different so SDMAX is set to infinite
636_Wilson_2018	1995		0,0418	0,049099	LETTER - LSD	4	15	
636_Wilson_2018	1996		0,005308	0,019905	LETTER - LSD	4	15	
676_Zhang_et_2018	May		0,132306	0,220509	LETTER - LSD	10	36	
676_Zhang_et_2018	September		0,066153	0,253586	LETTER - LSD	10	36	

Studies ID	*Set*	*SDValue*	*SDMin*	*SDMax*	*Method*	*n*	*DFE*	*Notes*
711_Kumar_et_2023	0-15cm		0,031562	0,038477	LETTER - Duncan	3	10	
711_Kumar_et_2023	15-30cm		0,045765	infinite	LETTER - Duncan	3	10	All means are not significantly different so SDMAX is set to infinite
711_Kumar_et_2023	30-60cm		0,008245	0,010259	LETTER - Duncan	3	10	
28_Basak_et_2012	after maize	0,034262	0,031407	0,037117	LSD	3	14	
28_Basak_et_2012	after wheat	0,029123	0,028837	0,029408	LSD	3	14	
458_Prakash_et_2016	0-7,5cm		0,034483	0,064312	LETTER - Duncan	3	10	
458_Prakash_et_2016	7,5-15cm		0,060464	0,057971	LETTER - Duncan	3	10	
708_Mourya_et_2023	15-30cm		0,006159	0,013601	LETTER - Duncan	4	21	a=>b reversed + errors
708_Mourya_et_2023	0-15cm		0,012954	0,013601	LETTER - Duncan	4	21	a=>b reversed + errors
708_Mourya_et_2023	30-60cm		0,006477	0,0068	LETTER - Duncan	4	21	a=>b reversed + errors
47d_Naklang_et_1999	0-10cm		0,061833	0,078696	LETTER - Duncan	3	12	1.22/1.26 a =>ab, CRD
350_Mandal_et_2020	0-15cm		0,03228	0,037977	LETTER - Duncan	4	15	
456_Polláková_et_2016	0-20cm		1,031085	1,766859	LETTER - LSD	3	6	
600_Viera_et_2007	Crops x5 - 333mM		0,714766	infinite	LETTER - Duncan	6	6	All means are not significantly different so SDMAX is set to infinite
443_Passos_et_2007	5-10cm - 33mM		0	0,13293	LETTER - Tukey	4	6	All means are significantly different so SDmin is set to 0
443_Passos_et_2007	15-20cm - 15,6mM		0	0,109812	LETTER - Duncan	4	6	All means are significantly different so SDmin is set to 0
443_Passos_et_2007	15-20cm - 33mM		0	0,242743	LETTER - Duncan	4	6	All means are significantly different so SDmin is set to 0
443_Passos_et_2007	5-10cm - 15,6mM		0,00578	infinite	LETTER - Duncan	4	6	All means are not significantly different so SDMAX is set to infinite

Supplementary material Table 2: Assignment of Reference Soil Group, soil clay content and soil pH.

Apart from total organic carbon content, soil type, clay content and pH were the variables most relevant for differentiating the studied soils and most systematically reported across studies. In cases where they were not reported, values were estimated using the logic given below. For each variable, values were also grouped to facilitate statistical analysis of the co-variable effects on POXC.

Database users should be aware that despite our efforts, there remains some uncertainties regarding the harmonisation of those added variables. Authors did not systematically provide all the details regarding the measurements (soil depth, protocols, etc.) and those added variables were mostly related to soil initial conditions and not often assessed across all treatments (pH could be influenced by the input treatments for instance). For total organic carbon, a complementary data extraction would be needed to harmonise data and go beyond the initial soil conditions to be consistent with the POXC measurement resolution.

"Best RSG (WRB)" is the Reference Soil Group from the World Reference Base, chosen as follows

RSG from paper if given, or if not:

RSG interpreted from soil classification or description in paper, or if not possible:

RSG from global map unit based on the experimental site coordinates

"Best clay" is clay content (%), chosen as follows:

Clay content for top layer from paper if given (median if range given), or if not:

Clay content for top layer estimated from texture (median of range for that texture group) from paper if given, or if not:

Clay content for 0-20 cm from global map unit

"Best pH" is pH (by whatever method used), chosen as follows:

pH for top layer from paper if given, or if not:

pH for 0-20 cm from global map unit (pH water)

Groups

pH group: 1 (pH<6), 2 (pH 6-7), 3 (pH>7)

Clay group: 1 (<20%), 2 (20-30%), 3 (>30%)

RSG group:

RSG	*RSG description*	*Group*	*Group description*
HS	HS Histosol (with thick organic layers)	1	**Soils with thick organic layers**
AT	AT Anthrosol (with long and intensive agricultural use, often altered to enhance fertility)	2	**Soils with strong human influence**
TC	TC Technosol (containing significant amounts of artefacts)	2	**Soils with strong human influence**
CR	CR Cryosol (permafrost-affected)	3	**Soils with limitations to root growth**
LP	LP Leptosol (thin or with many coarse fragments)	3	**Soils with limitations to root growth**
SC	SC Solonchak (high concentrations of soluble salts)	3	**Soils with limitations to root growth**
SN	SN Solonetz (with a clay-enriched subsoil with high concentrations of exchangeable Na)	3	**Soils with limitations to root growth**
VR	VR Vertisol (high contents of shrink-swell clays, alternating wet-dry conditions)	3	**Soils with limitations to root growth**
AN	AN Andosol (with allophanes and/or complexes of Al and organic matter)	4	**Soils distinguished by Fe/Al chemistry**
FR	FR Ferralsol (dominance of kaolinite and oxides)	4	**Soils distinguished by Fe/Al chemistry**
GL	GL Gleysol (groundwater dominated, underwater or in tidal areas)	4	**Soils distinguished by Fe/Al chemistry**
NT	NT Nitisol (low-activity clays, P fixation, many Fe oxides, strongly structured)	4	**Soils distinguished by Fe/Al chemistry**
PL	PL Planosol (stagnant water, abrupt textural difference)	4	**Soils distinguished by Fe/Al chemistry**
PT	PT Plinthosol (accumulation and redistribution of Fe)	4	**Soils distinguished by Fe/Al chemistry**
PZ	PZ Podzol (subsoil accumulation of organic matter and/or oxides)	4	**Soils distinguished by Fe/Al chemistry**
ST	ST Stagnosol (stagnant water, no or only moderate textural difference)	4	**Soils distinguished by Fe/Al chemistry**
CH	CH Chernozem (very dark and well-structured topsoil, secondary carbonates)	5	**Pronounced accumulation of organic matter in the mineral topsoil**
KS	KS Kastanozem (dark topsoil, secondary carbonates)	5	**Pronounced accumulation of organic matter in the mineral topsoil**
PH	PH Phaeozem (dark topsoil, no secondary carbonates (unless very deep), high base status)	5	**Pronounced accumulation of organic matter in the mineral topsoil**
UM	UM Umbrisol (dark topsoil, low base status)	5	**Pronounced accumulation of organic matter in the mineral topsoil**
CL	CL Calcisol (accumulation of secondary carbonates)	6	**Accumulation of moderately soluble salts or non-saline substances**
DU	DU Durisol (accumulation of, and cementation by, secondary silica)	6	**Accumulation of moderately soluble salts or non-saline substances**
GY	GY Gypsisol (accumulation of secondary gypsum)	6	**Accumulation of moderately soluble salts or non-saline substances**
AC	AC Acrisol (low-activity clays, exchangeable Al > exchangeable base cations)	7	**Soils with clay-enriched subsoil**
AL	AL Alisol (high-activity clays, exchangeable Al > exchangeable base cations)	7	**Soils with clay-enriched subsoil**
LV	LV Luvisol (high-activity clays, exchangeable base cations ≥ exchangeable Al)	7	**Soils with clay-enriched subsoil**
LX	LX Lixisol (low-activity clays, exchangeable base cations ≥ exchangeable Al)	7	**Soils with clay-enriched subsoil**

RSG group:

RSG	*RSG description*	*Group*	*Group description*
RT	RT Retisol (interfingering of coarser-textured, lighter-coloured material into a fine stronger-coloured layer)	7	**Soils with clay-enriched subsoil**
AR	AR Arenosol (very sandy)	8	**Soils with little or no profile differentiation**
CM	CM Cambisol (moderately developed)	8	**Soils with little or no profile differentiation**
FL	FL Fluvisol (stratified fluviatile, marine or lacustrine sediments)	8	**Soils with little or no profile differentiation**
RG	RG Regosol (no significant profile development)	8	**Soils with little or no profile differentiation**

Supplementary material Table 3: Check list of FAIR principles applied to the database, based on https://www.go-fair.org/fair-principles/

Findable	*Accessible*	*Interoperable*	*Reusable*
F1. **(Meta)data are assigned a globally unique and persistent identifier**<br>DOI generated by the Dataverse: https://doi.org/10.18167/DVN1/78A2II	**A1. (Meta)data are retrievable by their identifier using a standardised communications protocol**<br>Via the Dataverse Project, data are indexed in meta motors (OpenAire, re3data, Datacite etc.)	**I1. (Meta)data use a formal, accessible, shared, and broadly applicable language for knowledge representation.**<br>Data are provided in CSV, which is an Open-source format used for the interchange of database information between machines of two different architectures. The plain-text character of CSV files largely avoids incompatibilities such as byte-order and word size.	**R1. (Meta)data are richly described with a plurality of accurate and relevant attributes**<br>Detailed metadata were provided based on a mandatory harmonised CIRAD template and completed with further information including: title in 3 languages, controlled keywords and topic classification, all details on data producers and funding information, etc.
**F2. Data are described with rich metadata**<br>Metadata provided (R1) enable efficient research by key words.	**A1.1 The protocol is open, free, and universally implementable**<br>Dataverse protocols are free: HTTPS, OAI-PMH	**I2. (Meta)data use vocabularies that follow FAIR principles**<br>Key words were taken from controlled vocabulary sources:<br>http://aims.fao.org/aos/agrovoc<br>https://agrist.cirad.fr/agrist-thema	**R1.1. (Meta)data are released with a clear and accessible data usage license**<br>CC BY 4.0
**F3. Metadata clearly and explicitly include the identifier of the data they describe**<br>Detailed metadata were provided based on a mandatory harmonised CIRAD template and reviewed by Dataverse correspondants.	**A1.2 The protocol allows for an authentication and authorisation procedure, where necessary**<br>Authentication through Cirad SSO. Metadata and quote remain accessible without any authentication.		**R1.2. (Meta)data are associated with detailed provenance**<br>Origin of the dataset, versioning, and tracked changes are all made available.
**F4. (Meta)data are registered or indexed in a searchable resource**<br>Cirad Dataverse platform is an Open searchable resource.	**A2. Metadata are accessible, even when the data are no longer available**<br>Citation and metadata are fully public, and remain available even if the dataset becomes unavailable.		**R1.3. (Meta)data meet domain-relevant community standards**<br>Key words were taken from controlled vocabulary sources specific to the domain :<br>http://aims.fao.org/aos/agrovoc<br>https://agrist.cirad.fr/agrist-thema
